# A community-based co-designed genetic health service model for Aboriginal Australians

**DOI:** 10.1371/journal.pone.0239765

**Published:** 2020-10-29

**Authors:** Imogen Elsum, Libby Massey, Callum McEwan, Desiree LaGrappe, Emma Kowal, Ravi Savarirayan, Gareth Baynam, Misty Jenkins, Gail Garvey, Margaret Kelaher

**Affiliations:** 1 Centre for Health Policy, University of Melbourne, Melbourne, Victoria, Australia; 2 MJD Foundation, Alyangula, Northern Territory, Australia; 3 James Cook University, Townsville, Queensland, Australia; 4 Alfred Deakin Institute, Melbourne, Victoria, Australia; 5 Victorian Clinical Genetic Services, Melbourne, Victoria, Australia; 6 Genetic Services of Western Australia, Perth, Western Australia, Australia; 7 Walter and Eliza Hall Institute of Medical Research, Melbourne, Victoria, Australia; 8 Menzies School of Health Research, Darwin, Northern Territory, Australia; University of South Australia, AUSTRALIA

## Abstract

**Background:**

Aboriginal and Torres Strait Islander people experience a greater burden of disease and die younger than non-Indigenous Australians, with Aboriginal people living in remote areas of the Northern Territory of Australia having the lowest life expectancy estimates. Despite a high burden of chronic disease among Aboriginal and Torres Strait Islander people, access to specialist health services remains low and models of care that increase engagement, may improve health outcomes.

**Methods:**

We describe client and staff perspectives of a model of clinical genetics services provided by the MJD Foundation (MJDF) in geographically and culturally complex contexts within the Northern Territory of Australia. We seek to understand the MJDF model’s success in supporting Aboriginal families with the familial, neurodegenerative condition Machado-Joseph disease and how it could be applied in the provision of other specialist services. Thematic analysis was undertaken on semi-structured interviews with primary health care staff (n = 2), Non-Aboriginal MJDF Staff (n = 7) and Aboriginal MJDF Clients / Community workers (n = 13).

**Results:**

Four key themes regarding the MJDF model of service delivery were identified with the service being; 1) client led 2) accepting of various understandings of genetic disease causation 3) focused on relationships, continuity and trust between the service provider and the clients, and 4) committed to incorporating an inclusive whole-of-family practice. The MJDF model takes a community-based, person-and family-centred approach to successfully deliver effective specialist genetic health services in remote community settings. We propose that these approaches have broad application in the future design and delivery of specialist health services particularly in culturally complex settings.

## Introduction

Whilst individually rare, the 6–10,000 known rare monogenic disorders make these genetic diseases collectively common. Sixty percent of Australians develop a condition (of varying severity) with a genetic background at some stage in their lives [1] and globally more than 400 million people are living with a rare disease, warranting a global public health agenda [2]. Genetic diseases are clinically heterogeneous; highly varied symptoms exist for each condition and the needs for those living with each individual condition are similarly diverse. People with genetic and rare diseases often face substantial challenges including delayed diagnosis, difficulties accessing appropriate health services such as genetic health services and difficulties in accessing appropriate treatment or support [3].

In Australia, Aboriginal and Torres Strait people have a higher incidence of some genetically determined conditions [4, 5]. Despite demand [6, 7] their overall utilisation of specialist services is 43% lower than for other Australians [8]. This lower utilisation reflects in part the challenges faced accessing specialist services in remote areas where a greater proportion of the population is Aboriginal [9]. Specialist services, such as genetic services predominantly operate within urban hospital settings and remote patients are frequently required to travel long distances to access these services and there is chronic underutilisation of outreach programs [9]. These issues are amplified for clinical genetic services as most referrals derive from medical specialists rather than from primary health care health providers [10].

In Australia, achieving equitable access to genetic services is fraught with difficulties related to large geographical distances, scarcity of appropriately skilled staff and the infrequent and often isolated distribution of genetic diseases [11, 12]. Difficulties are amplified when cultural aspects that may influence or limit engagement with genetic health services are also considered [13]. Services deemed culturally competent consistently achieve higher engagement from Aboriginal and Torres Strait people than those which do not [14, 15]. To be effective, a culturally competent service takes into consideration and respect, the culture, values and traditions of the individual accessing the service [15]. In Australia, these approaches can be found in Aboriginal community-controlled health services (ACCHS) and also observed in the cultural safety improvements of some mainstream health services [16]. Use of these approaches in the provision of specialist services to date is limited [17].

Improving the cultural safety of specialist services is challenging in clinical genetics. These services are under-utilised by Aboriginal and Torres Strait people and the diseases detected are often rare. Consequently, service change is not typically demand driven at the individual disease level. However, the high concentration of the disabling genetic neurodegenerative condition Machado-Joseph disease (MJD), otherwise known as Spinocerebellar Ataxia Type 3, in remote north Australian Aboriginal communities has resulted in a unique situation where a collaborative community-based model of delivering specialist health services has developed in response to high local demand.

### Machado Joseph Disease and the MJD foundation

MJD is an autosomal dominant condition, either gender can be affected and individuals who are affected have a 50% chance of passing it to their children- rising to 75% if both parents are affected [18]. In small and geographically isolated Aboriginal communities in northern Australia, the prevalence of MJD is now one of the highest in the world providing a unique opportunity to co-design genetic services with the local Aboriginal communities [19]. MJD devastates families. Mean survival time after onset is 21 years, much of this lived with very high support needs [20]. Anticipation, a challenging characteristic of the disease results in signs and symptoms becoming more severe and appearing at an earlier age as the disease is passed from one generation to the next [21, 22]. As a result of genetic anticipation, concurrent generational manifestation within families is common, compromising care within family settings. Children who might otherwise be able to care for parents severely disabled by the disease are themselves disabled, sometimes more rapidly than their parents. In remote Aboriginal communities, this is exacerbated by limited local health and disability services and rudimentary or non-existent access to preventative, rehabilitative, therapeutic, allied health interventions, health education and genetic services.

The Machado Joseph Disease foundation (MJDF) was established in 2008 to provide locally accessible, appropriate and sustainable care. Initially based on Groote Eylandt, the MJDF’s services have expanded over the last decade to other remote Aboriginal communities across the Northern Territory and Far North Queensland. As a community derived service, MJDF supports are client driven and have developed iteratively in response to local conditions, and at the behest of families. MJDF services are inclusive of the whole family impacted by MJD, including those ‘at risk’ of developing the disease, caregivers and extended family. Programs comprise specialist disability support and education, genetics, social and emotional wellbeing programs and case management. All programs are delivered within the community, by allied health, social work and nursing professionals who work in tandem with Aboriginal community workers (ACWs). The model is client led and places a premium on conducting all activities within the cultural expectations and norms of local communities. Relationships between clients and MJDF staff, facilitating opportunities for clients to spend time ‘on Country,’ that is to say on ancestral lands, and local delivery are key principles of MJDF’s service delivery. Supports provided are in direct response to MJDF’s clients’ and families’ individual requirements.

### MJDF genetic services

Improving access to clinical genetic care, genetic education and counselling emerged as early priorities after the MJDF was established. These needs had previously proven difficult to address consequent to long term national scarcity of genetics professionals (clinical geneticists and genetic counsellors) and the remote location of the families.

Unsurprisingly therefore, Aboriginal families with MJD engaged minimally with genetics professionals prior to 2010, when a consultant clinical geneticist offered his expertise to MJDF as a volunteer. His subsequent community-based consultations with families framed best practice engagement for those at risk of MJD. The prioritised services are characterised by: 1) local delivery (not outreach) 2) appointments conducted in the person’s first or preferred language or with language and cultural interpretation 3) a flexible format able to accommodate lengthy, gender-specific and group patient interactions and 4) a preference for long lead times from first clinical contact to molecular testing.

Present-day MJDF counselling services are provided in each community by a visiting accredited genetic counsellor who works in partnership with MJDF community-based ACWs and allied health Managers of Community Services. Local ACWs attend all sessions and assist family and wider community understanding of the disease by the development of educational tools describing the genetic transmission of MJD in local languages. These resources are iteratively modified to accommodate world view differences and knowledge gaps.

For the ongoing continuation of the MJDF clinical genetics program, it has been important to accommodate a preference for single gender, communal consultation sessions conducted within the person’s home community, often in an informal setting. The western biomedical, clinical orientation to individualised care and privacy and the security of information exchange likewise must be tailored to the relevant world view of collectivist culture held by many MJD clients [23]. To comply with these views and also conform to existing ‘best practice’ ethical and clinical protocols, management of information must be carefully negotiated on a case-by-case basis to ensure the appropriate family members are consulted and to avoid local misunderstanding and shame over the transmission of disease through the family [24]. Close and ongoing engagement with the family through the ACW is vital to managing and maintaining expectations throughout the process of genetic counselling.

The MJDF has prioritised employment of Aboriginal people affected by the disease as ACWs as well as continuity of non-Aboriginal staff to maximise long-term relationships within the communities. Developing these relationships and increasing the number of ACWs is more easily achieved in a local setting with a high Aboriginal population such as Groote Eylandt (98.8% Aboriginal) [25]. However, the MJDF model has resulted in improved engagement of Aboriginal people with clinical genetics services and provides a case for altering the paradigm of how specialist services are delivered.

We explore what aspects of the MJDF model are central to the improved engagement with clinical genetics services and submit that Aboriginal Australians uptake of specialist health services could be improved to better meet community needs through the provision of accessible, culturally responsive, client led, and flexible approaches. An in-depth exploration of the client and staff perspectives from the MJDF will be presented and elements of service provision identified that could be applied to other specialist services that aim to operate in geographically and culturally complex contexts.

## Methodology

### Study design

A case study approach was used in this study, the case defined as the MJDF model with select settings in which the model operates, providing real-life context. Flexible approaches were taken to ensure that the research was responsive to participants and respectful of cultural values [26]. The interview guides were designed to capture perspectives from the different components of the health system interacting with the MJDF model, and therefore included end-users (MJDF clients), service providers (MJDF staff), and primary care workers. Ethical approval was granted by the Human Research Ethics Committee (HREC) of the Northern Territory (NT) Department of Health and Menzies School of Health Research (HREC 2018–3075) and The University of Melbourne (HREC 1648489.4). Permission and support to conduct this research on Groote Eylandt was granted by the Anindilyakwa Land Council (ALC). Funding was provided by a Lowitja Grant and a National Health and Medical Research Council Partnership Grant in which, MJDF a partner in the research, provided financial and in-kind support.

### Setting

**Fig 1:**
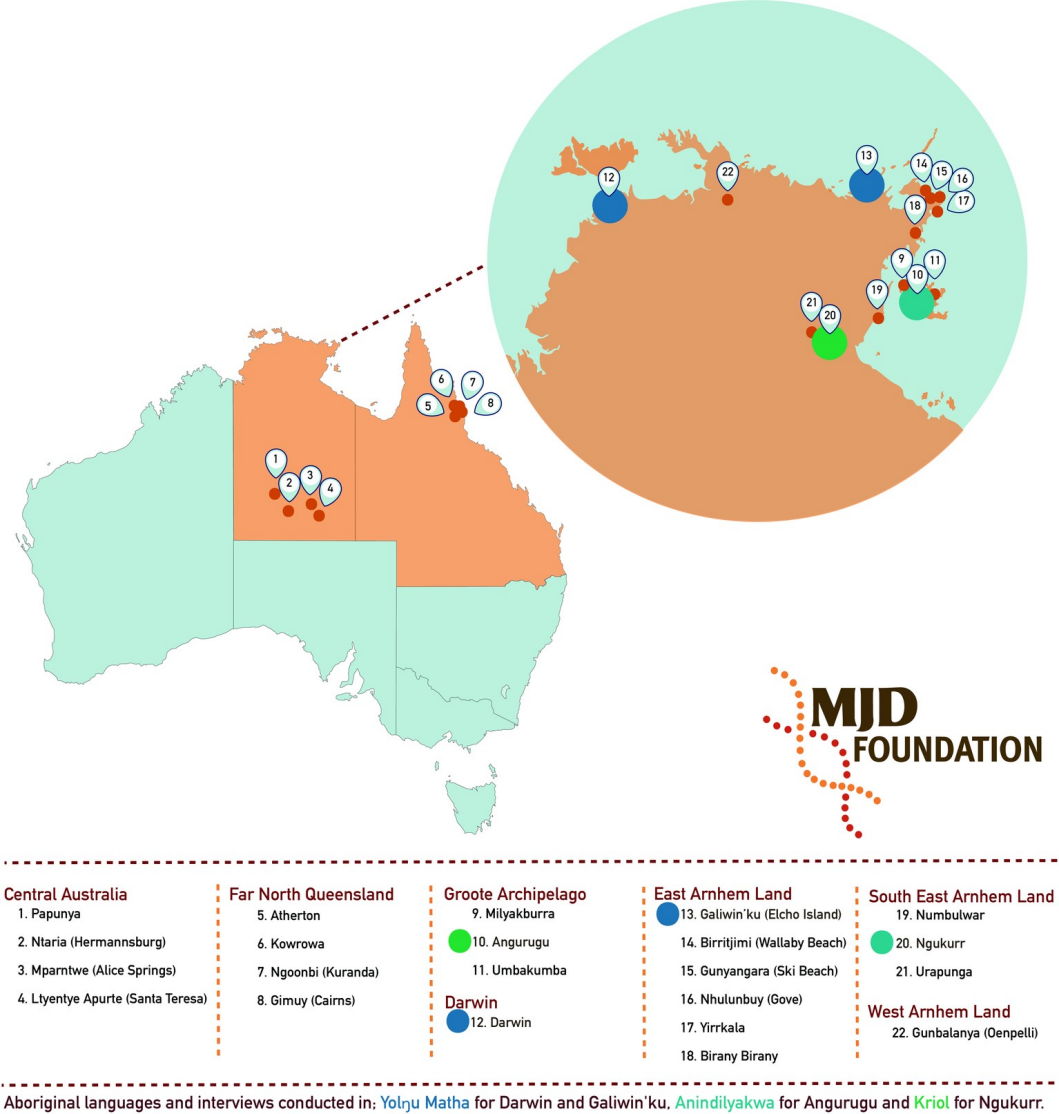
Location of MJDF services and interviews.

In consultation with the MJDF, research settings were selected that capture three Aboriginal language groups; Yolngu, Anindilyawka and Kriol, in communities where the MJDF services are embedded (Fig 1). Yolngu are the Traditional Owners of north-eastern Arnhem Land in the Northern Territory of Australia [27]. The Yolngu people who participated in this study were originally from Galiwin’ku; however, as many of the MJDF clients have relocated to Darwin in order to access adequate services, interviews with Yolngu clients were conducted in Darwin. Anindilyakwa is the language spoken by the Warnindilyakwa people, the Traditional Owners of Groote Eylandt and Bickerton Island in the Gulf of Carpentaria in the Northern Territory of Australia [28]. Interviews were conducted at Angurugu on Groote Eylandt. The Traditional Owners of the land around Ngukurr include the Alawa, the Binbingka, the Marra, the Ngarnji, the Wilangarra and the Yanyuwa people. There are several Aboriginal languages spoken in Ngukurr however, the universal language is Kriol and Aboriginal English is also widely spoken [29]. Kriol speaking participants were interviewed at Ngukurr. Non-Aboriginal MJDF staff members often work across these language groups and were interviewed in community. Primary care health staff were interviewed at Angurugu, Groote Eylandt.

### Participants

Participants were purposively selected to capture different perspectives that interact with the MJDF model and categorised into one of four groups: 1) Primary health care staff (n = 2) based at Angurugu, Groote Eylandt where the MJDF service originated, 2) non-Aboriginal MJDF staff (n = 7), 3) Aboriginal MJDF dual clients/community workers (n = 5) and 4) Aboriginal MJDF clients (n = 8) (Table 1). Five of the thirteen Aboriginal MJDF clients interviewed were employed as MJDF community workers. In the thematic analysis, all clients were grouped together to capture the aggregated perspectives of MJDF clients.

**Table 1:** Interview participants.

Participant	Language	N	Role
Primary health care staff	English	2	Primary health care staff (n = 2)
Non-Aboriginal MJDF Staff	English	7	Physiotherapist (n = 1), genetic counsellor (n = 1), social worker (n = 1), occupational therapist (n = 2), nurse (n = 1), community worker (n = 1)
MJDF Clients/MJDF Community Workers	Yolngu	5	Individuals with a clinical or genetic diagnosis of MJD (n = 10), individuals who were carers and had either been tested and did not carry the MJD gene (n = 1) or were at risk and had not undergone genetic testing (n = 2)
	Anindilyawka	4	
	Kriol	4	

All participants were aged over 18 years and able to provide informed consent. Participants that were MJDF clients were selected to ensure a wide range of experiences including those who had genetic testing and those who had not. Individuals known to the MJDF services were initially approached by MJDF service manager LM and invited to participate. Interested participants were then approached by researcher IE, to discuss the study and seek informed consent.

### Data collection and analysis

In the scoping phase, researcher IE visited MJDF communities with the aim of developing an understanding of MJDF services and building relationships in each community. This led to researcher IE conducting face-to-face, semi-structured interviews (n = 22). All interviews were conducted between November 2017 and July 2018. Participants had the opportunity to select the interview location, communicate in their preferred language and receive the transcript of their own interview for review and comment. Participants were given the option of having family members or a support person with them during the interview. Tailored interview guides were developed for each participant group; Aboriginal MJDF clients (the same guide used if they were also a community worker), non-Aboriginal MJDF staff and primary care health staff. Aboriginal MJDF clients, including those who were MJDF community workers, are collectively referred to as MJDF clients throughout this paper however, it is noted in the source of any quotes if the individual is also a community worker due to the different perspective this brings. Interviews with MJDF clients covered stages of a typical journey for patients utilising clinical genetic services; these were home/community, visits to a primary care clinic, a clinical genetics appointment and follow up care. MJDF staff were asked to reflect on the role of genetic education and testing in the holistic MJDF model before being asked more broadly about the different dimensions of accessibility within the MJDF model of care. Primary health care staff were invited to share their experiences in providing services to MJDF clients and the strengths and weaknesses of the MJDF model for remote Aboriginal populations more generally. Where necessary interpreters were used. Interpreters for the Yolngu and Anindilyawka language groups were connected to the MJDF and had existing relationships with participants. Despite the potential for a conflict of interest, existing relationships were deemed to be critical to ensure a genuine dialogue. Observations and reflections recorded in field notes were also included as data.

Interviews were audio recorded and transcribed using Rev transcription services (www.rev.com). Transcripts were quality checked by IE with assistance from LM when necessary. NVivo software (QSR International’s NVivo 11) was used to manage transcripts and for thematic analysis. Observational field notes were manually reviewed to test validity of interview data and add contextual information around the history of the MJDF services in each community. Interview transcripts were coded by a single researcher (IE) and analysis undertaken by linking identified core themes to principles aligned with service provision, namely client led and responsive with a focus on culture, communication and workforce [26]. Review of data throughout analysis was undertaken by researchers MK and LM.

## Results

Emergent themes describing the MJDF model and the care provided, were similar across the three Aboriginal languages, Yolngu, Anindilyawka and Kriol. Analysis from all settings corroborated the importance of four key intertwined approaches to service delivery. MJDF service delivery; 1) was client led; 2) incorporated a shared understanding, where a variety of explanations for disease causation were accepted by the MJDF, not only those aligning with a biomedical model; 3) involved relationships, continuity and trust between the MJDF as service providers and their clients, and 4) incorporated a family approach. Identified approaches closely align with principles of participatory healthcare and a person-and family- centred approach, namely treating clients with dignity and respect and communicating information to enhance understanding among clients so they are empowered to be an active participant in decision-making around their own health.

### Theme 1: Client led

The guiding MJDF organisational principle of developing and maintaining a client led service was evident in the interviews with non-Aboriginal MJDF staff members, MJDF community workers and the field observations. There was implicit and explicit acknowledgment that to maximise engagement in service activity there must be genuine input and directions from the client from the very beginning.

*“Visiting people like doing research and* ***finding out what they need*** *and that they need more help*. *Wasn't easy*. *It used to make me sad because I was come there to ask my own families these things*.”

*Quote*: Aboriginal MJDF dual client/community worker

*“We listen to what they want us to do*. *What they want*, *and then try to find a way to facilitate it*, *and I think that's unique in that a lot of [non-MJDF] programs that are developed*, *are developed from an external perspective and they're imposed on a community and they may or may not be appropriate*. *Sometimes they are*, *and sometimes they're not*, *whereas we're actually coming at it from a*. . . .*” we’ve been working with people inside the community with MJD*, *these are the things that we've done there*. ***These are the things that they've wanted”***.

*Quote*: *non-Aboriginal MJDF staff*

As these quotes from an Aboriginal MJDF client/community worker and a non-Aboriginal MJDF staff member illustrate, there was recognition by MJDF that for the organisation to succeed and make an impact on the lives of those it was set up to help–that is, the people impacted by MJD—the directions and activities of the organisation must come from them.

### Theme 2: Shared understanding

The emphasis the MJDF places on incorporating principles of learning and respecting Aboriginal culture and knowledge was a common feature throughout the interviews. This included respect and consideration for different worldviews and for different understandings of why and how a disease may come ‘down the family line’. There was a view expressed by both primary health care and MJDF staff, that the concept of genetic transmission was well understood by MJDF clients, even if alternative explanations to explain why the disease was in the family were simultaneously held. A view often expressed by health professional staff was that the MJDF clients understood that MJD was a genetic disease, an illness that came down the family line because of a faulty gene, yet concurrently held onto other beliefs such as the existence of a family curse. There was a shared understanding among MJDF clients and staff that these views could be held concurrently without one being more valid than the other.

*“There is a client who I am really close to on Groote Eylandt and he has got a really good understanding of MJD*. *He has MJD*, *he has been symptomatic for quite a while*, *he has probably been educated as much about MJD as any other client that we have at the moment*, *but he still believes whole heartedly that it's a curse and that's just his cultural belief and view*. *I don't try to push the envelope or convince him otherwise*.”

*Quote*: *non-Aboriginal MJDF staff*

Across all communities engaged with the MJDF, there were attempts to understand why some families were affected by MJD, yet not others. In this research, some participants reflected on the ancestral lineage and wondered why the family was selected to carry the disease.

*“One old man in particular………*. *he'd say to me*, *"I don't like your story*.*" That was because he had inherited his disease from his mother*, *and in his culture*, *you get your family's story from your father*. *For him to be told that he inherited the disease from his mother when he was supposed to inherit*, *pretty much anything that was important from his father*, *made him really uncomfortable*, *so he didn't like that story*. *He definitely had an intellectual conversation*, *that happened*, *and he caught on really quickly that all of his kids were at risk and he'd tell them and his grandchildren*, *but he didn't like it*. *I think it's a really complicated space*.”

*Quote*: *non-Aboriginal MJDF staff*

The MJDF promotes a culture of respect and strong relationships between staff and clients through their “two way” approach to service delivery. This is reflected in the inclusive approach taken by staff to people’s different views and beliefs including those relating to inheritance.

*“Not quite blood*, *but something inside*, *and that's the one that carries the sickness*”.

*Quote*: Aboriginal MJDF dual client/community worker

*“When I learned and understood about that*, *then I knew that the disease*, *I knew that we can't spread to other people*, *but it's inside*, *inside our body*. *It's in our blood that maybe father has or even a mother*. *And pass it to their children……………Learning and hearing from several people*, *then I understood what genetics was because we can say it is inside our body*.”

*Quote*: Aboriginal MJDF dual client/community worker

MJDF clients were acutely aware of the familial nature of MJD and spoke confidently when tracing how the illness came ‘down the family line’ suggesting that associations with the disease were strongly tied to their family story rather than the biological mechanisms of disease. Blood was frequently referred to when discussing the pattern of inheritance. Some associated the English word “genetics” with a familial illness without distinguishing different types of illnesses. By incorporating a shared understanding, the MJDF allows for the biological mechanisms to be understood without compromising cultural values or beliefs.

### Theme 3: Relationships, continuity and trust

From a non-Aboriginal MJDF staff and Aboriginal MJDF community worker perspective, the importance of building strong sustainable relationships with community members and clients was vital. It was viewed as an important way to build trust and thus facilitate meaningful conversations. Intrinsic to this trust was understanding the history of social and economic disadvantage experienced by many community members and the incredible hardships faced. There was an embedded understanding amongst the non-Aboriginal MJDF staff of the incremental nature of building respectful and trusting relationships with Aboriginal community members.

*“So because I myself didn't understand*, *but just the same that I was interested in learning*, *looking for a way that I could understand this disease and to help my family*. *Started working with [MJDF co-founders]*. *Cos from them I learned lot of things and they learned from me*, *too*, *as we were going on the journey and work together about this disease*. *What I didn't understand about genetic thing and kept on working because [MJDF co-founder] was on my side all the time*.”

*Quote*: *Aboriginal MJDF dual client/community worker*

The importance of having continuity of engagement emerged repeatedly and this was often intertwined with trust and relationships. There was a clear sense that non-Aboriginal MJDF staff felt it was very important for them to be able to provide a stable support network for the MJDF clients in communities where high health care-staff turnover in mainstream services is common.

*“Once you build trust with the family then you know that means that you are in with the whole family and that people are willing to talk to you and that's what I think sets us aside from a lot of other providers*. *Local people are just sick and tired of having to explain their story to strangers over and over again*.”

*Quote*: non-Aboriginal *MJDF staff*

The importance of continuity of care was reflected in the perspective of the MJDF clients who described a reluctance to share information with health professionals with whom they do not have an ongoing relationship. It was also alluded to in the hesitancy in talking about MJD with the staff at their local primary health clinic.

*“The ones I know*, *I can talk to them*. *But new ones*, *when they come in*, *I won’t talk to till I know them*”

*Quote*: Aboriginal MJDF dual client/community worker

Interviews with the non-Aboriginal MJDF staff highlighted that along with placing importance on relationships, it was acknowledged that these relationships will not develop overnight but take time.

*“They're getting to know* [genetic counsellor]. *It's been*, *I think it's been two years*, *but it takes a while*. *Relationships are everything*”.

*Quote*: non-Aboriginal *MJDF staff*

Having a presence in the community was seen not only to catalyse the development of these relationships but also to allow opportunistic ‘consultations’ or genetic education sessions when a client expresses an interest or feels they are ready.

### Theme 4: A family approach

All 13 Aboriginal MJDF clients interviewed mentioned the importance of family. It was viewed as an important source of support and comfort and helped them deal with MJD. Most clients stated that, when discussing their experiences with MJD, they preferred to talk as a family group. This was for support and also seemed to be a way of showing that there is no shame in the disease. It should be noted however, that although the vast majority preferred to discuss their MJD experiences in a group, two clients (15%) did specify that they would prefer to talk one-on-one about MJD to health professionals. Some stated that they don’t talk to anyone about MJD except their family. These findings illustrate the importance of client led interactions as well as continuously listening and checking in with clients.

*“Well*, *my sister*. *I never talk to my brothers because there's a culture*, *that I'm not allowed to sit next to my brother and talk*. *I used to sit with my sister*, *even my niece and even to my nephews*.”

*Quote*: *Aboriginal MJDF dual client/community worker*

*“No*, *not really*. *Because I don't talk about the MJD*, *I don’t tell anyone about this disease*. *Only family*.“

*Quote*: *Aboriginal MJDF client*

The importance of these family discussions, whether in the presence or absence of non-Aboriginal MJDF staff, was viewed as a very important aspect of the organisation. These informal discussions amongst family in the communities were significant in building capacity amongst the clients and in creating a sustainable body of knowledge.

*“what I think is really lovely and empowering and helpful for people*, *is when people within their family that have been through the process before can talk to them about that*, *and be involved in those conversations and help make people feel more comfortable and confident that this is something that they can do*”.

*Quote*: non-Aboriginal *MJDF staff*

There was a recognition by MJDF and primary care staff that the genetic nature of MJDF and the impact it had on all family members meant there was a strong feeling of serving families rather than individuals.

*“we've had very strong directive from our families*, *that it is a communal thing*. *It's not something that affects individuals*, *it's something that reflects the whole family*”.

*Quote*: non-Aboriginal *MJDF staff*

The MJDF was aware that there are specific people in the family with whom it is important to engage early in the process of establishing relationships. This factor was important when considering whom the MJDF initially reached out to in the community and who was involved on an ongoing basis. The collective decision-making culture that is strong in these communities was respected and non-Aboriginal MJDF staff followed the guidance of community members.

*“Input and that guidance from family and specific family members have different roles*. *There are some very key people in the family that are really important*, *who are involved in decision making*.”

*Quote*: non-Aboriginal *MJDF staff*

Aligning with the importance placed on family structures, the Aboriginal MJDF clients viewed learning and contributing to the recording of their family tree (pedigree) very positively. Although some felt upset by the reminder of how many people in their family have died from the disease, it was viewed as a good way to tell their family’s story and some spoke about sharing their family tree, so others could learn from their story. In contrast, most clients had limited recall of the videos used as part of the genetic education sessions, nor recall the content. Collectively this suggests that there is a deep appreciation of MJD as a familial disease and although there is an embedded understanding of whom in the family line has been affected, the mechanism of disease is not considered to be as important for most of these family members.

*“It [family tree] makes me feel good because I sort of know my family from way*, *way back…… Makes me feel proud about them*”.

*Quote*: *Aboriginal MJDF dual client/community worker*

Whilst some people felt sad seeing how many people in their family had been impacted or died from MJD, others were quite neutral in their response, agreeing that recording the family tree was important without getting too emotionally caught up in it.

*“Well it’s normal*. *Everyone has their own family sickness and you have to all be there for each other*.”

*Quote*: *Aboriginal MJDF client*

Community workers found the family trees a good way of teaching others in their family about the inheritance of the MJD.

*“I feel happy when I see the family trees*, *my family’s tree*. *I tell them if they want to know about this disease*, *they could look at this chart*. *Look and know that it's there for them to learn*, *too*. *Not only us as a family*, *not as a client*, *but this disease that is for everyone if they want to come and ask for family trees*. *So it's here for them to see*.”

*Quote*: *Aboriginal MJDF dual client/community worker*

One individual who lives with MJD felt a strong sense of pride and desire to help others in their family who are impacted by MJD.

*“I'm the only one in my family that*… *I suppose you can say that I'm a person that I went to school and got my education*. *I understand both worlds*. *It makes me feel proud and stronger when I do something for my family*. *When I help my family go through this terrible disease…………*.

*So*, *that's where I feel that I'm the one who can do something with my family and help them out with this disease that's robbing my family of their lives and myself*. *I want to do something*. *I want to achieve something for my family and my kids*.”

*Quote*: Aboriginal MJDF dual client/community worker

Non-Aboriginal MJDF staff also viewed discussions about the family tree with clients and community members as an important way of opening up dialogue around MJD.

*“we will do things like pull out their identified family trees and use them to explain it*. *Different ways of kind of exploring our understanding of how MJD came to be in their family*, *and how it gets passed on*, *and what that means for them*”.

*Quote*: *non-Aboriginal MJDF staff*

This discussion creates a safe space where people can share any concerns, worries or questions they have about themselves or other family members. It also provides a forum to open up conversations about who else in the community may be at risk of, or affected by, MJD.

## Discussion

MJDF has played a pioneering role developing a model that provides appropriate, culturally informed clinical genetic services to Aboriginal communities. This study demonstrates that the model is oriented to person- and family- centred care and brings together key principles from the cultural safety literature, namely the existence of shared respect and knowledge between MJDF as the service provider, and the clients as the recipients, to overcome cultural power imbalances and provide an environment where clients feel safe and secure in their identity, culture and community whilst accessing services. The MJDF model; 1) is client led; 2) develops shared understanding where a variety of explanations for disease causation are accepted by the MJDF, acknowledging that the biomedical explanations and traditional cultural understandings can exist concurrently; 3) is predicated by relationships, continuity and trust between the MJDF as service providers and their clients, and 4) involves a family approach. The MJDF model stands in stark contrast to other models of specialist clinical service provision that are predominantly fly-in and fly-out outreach clinics and rely heavily on having a well-established and functional primary health care service in the community. The MJDF approach has developed in response to needs in regions with a high prevalence of a specific genetic condition, a ‘family’ disease necessitating a family-oriented response [30]. Their way of working, including cultural considerations is particular to the communities with which MJDF works. For these reasons, some elements of the model are not directly transferable to other settings, however there are many aspects that are translatable as discussed below.

The central characteristic of the MJDF model of service that emerged from this study is that it is fundamentally directed by the client’s expressed needs. This principle underpins the operations of the service model and directly or indirectly fed into all other themes that emerged in this research. Cultural safety enables ‘safe’ services to be defined by those who are the recipients of the service [31, 32]. This strongly aligns with the MJDF model with its emphasis on ensuring the services offered are based on client needs and preferences. Elements include giving a voice to the clients around where and when services are conducted. An example of this is the ‘on-country therapy’ program which emerged from clients expressing a clear preference for the exercise programs offered by MJDF being conducted on-country (on ancestral lands) where clients feel connected, comfortable and safe. Another is how the genetic education sessions are held in the communities, under trees or on verandas, and run as separate group sessions of men and women aligning with the expressed wishes of the clients themselves [33].

MJDF’s emphasis on placing the client’s needs at the centre of service provision, not only reflects values of cultural safety; it also conforms to models of person- and family-centred care [31]. Growing evidence suggests that this improves health outcomes and increases patient satisfaction [34]. This concept has also recently gained traction in genomics policy in Australia as evidenced by its inclusion as one of the five strategic priorities in the National Health Genomics Policy Framework 2018–2021 [35, 36].

The Australian health system historically privileges the Western biomedical model of health and remains focused on biomedical sciences and understanding the physiological causes of disease and illness [37, 38]. This philosophy differs fundamentally from the wholistic view of Indigenous cultures that incorporates physical, cultural, social and emotional wellbeing [17, 39, 40]. Traditional practices, beliefs and medicines are a part of daily life in many Aboriginal communities, however there is very little documented recognition or appreciation of the role they play in the individual’s and community’s health and wellbeing within biomedically-focused clinics [41, 42]. It has been recognised that strong partnerships between Aboriginal and mainstream Western biomedical organisations are integral to improving Aboriginal health outcomes in Australia however, there are significant cultural barriers and challenges [40].

The MJDF navigates this by ensuring their services are bicultural and integrate the perspectives of Aboriginal Australians into every level of their service, from the governance structures to the community services. This approach has strong similarities to two-way, bicultural practice frameworks that have been developed to facilitate a broader perspective when delivering services within Aboriginal communities in Canada [43]. The ‘two-eyed seeing’ framework discusses the importance of allowing equal contributions to diverse Aboriginal and non-Aboriginal worldviews such that no view dominates or undermines the other [44]. Likewise, a framework designed specifically for organisations delivering services to children and families in the Northern Territory highlights the importance of cultural safety and co-working models where there is a shared understanding of concepts that relate to the services and culturally respectful engagement [45].

To achieve shared understanding and cultural safety within health services, there must also be trust and strong relationships between health workers and consumers of the service. It was clear from both the interviews and observational data of the MJDF service, that relationships are a fundamental focus of the organisation. Focusing on strong relationships enables the organisation to implement co-working models between Aboriginal and non-Aboriginal people. These strong relationships promote cohesiveness and integration not just within the organisation but within the broader Aboriginal communities the MJDF work in. It also allows the service to extend cultural safety and incorporate cultural knowledge and practices into service delivery—considered as cultural congruence/integration [46]. The deep trust between MJDF clients and staff that has been built up over many years means that clients will extend their trust and respect to individuals external to the organisation who are trusted by MJDF staff. This means that when the MJDF service needs to bring in specialist expertise such as neurologists, there is a more rapid acceptance of these individuals due to an unspoken understanding and expectation that MJDF would only introduce people into the community if they adhere to the values held by the MJDF. When services or appointments cannot be held in the community, MJDF staff members provide practical support to clients to navigate appointments for mainstream services and ACCHSs.

Delivering appropriate services to Aboriginal Australians–particularly those in remote areas–has been plagued by long-standing challenges. Several studies have found that there are significant sustainability issues associated with current models of primary and specialty care [9, 25, 47]. Strategies solely relying on the provision of fly-in fly-out/drive-in drive-out specialist health services to remote communities have done little to address these disparities in health outcomes. Important deficits include issues related to the facilitation of culturally responsive care and workforce retention [9]. Adapting the MJDF approach to other clinical genetic services which are fly-in fly-out is challenging in the absence of strong relationships between the people and the communities. A way in which this might be achieved is by leveraging relationships with existing service providers and developing a local Aboriginal workforce. By employing a local Aboriginal workforce, MJDF negates these identified workforce deficits as MJDF community workers are already living in these areas and are innately familiar with cultural aspects that may limit service engagement. Building increased capacity of local Aboriginal workers is essential in remote areas where limited housing, facilities and peripheral services make it problematic for mainstream services to employ permanent staff [48]. This is particularly true for rare genetic disorders with intermittent distribution of potential clients across large geographic areas. In such instances, the relationships with local community organisations becomes integral to facilitate these interactions.

Aboriginal community-controlled health services follow a social model of health as opposed to a biomedical one, with a philosophy of providing wholistic, comprehensive care that is culturally informed [49]. Within this model of care, Aboriginal Australians feel more comfortable accessing health care and incremental improvements are being seen in the emotional, social and physical wellbeing of the population [50]. High numbers of Aboriginal staff and a lower overall staff turnover contribute to ACCHSs being more culturally safe alternatives to mainstream health services. A 2016 study in which over 120 Aboriginal Australians were interviewed, found that having health care consultations in a space where they felt ‘safe’ was crucial to ongoing engagement with the health system [51]. In remote areas, the services offered by ACCHSs centre on primary care, preventative care and support services with access to specialist health services generally coordinated through outreach models. Due to the nature of current models of delivery via outreach, specialist health services are unable to build sustained relationships with the local Aboriginal community. Working with organizations such as MJDF or the local ACCHS may facilitate the trust and relationships that are important to the recipients of their care. Actively involving the local Aboriginal community and developing strong relationships between service providers and the community is key to facilitating effective engagement [52, 53]. Strengthening the link between ACCHSs and clinical genetics services, or other specialist services, may lead to better service utilisation and engagement with Aboriginal Australians by providing these services in a setting they deem to be culturally safe [54].

The emphasis on a family approach that emerged in this study is congruent with the focus MJDF places on relationships. Governance and decision-making processes in Aboriginal communities revolve around leadership and family structures that often differ from non-Aboriginal communities [55]. Since inception MJDF has been guided by local Aboriginal community members to ensure that local structures are respected and followed. The autosomal dominant inheritance pattern of MJD means service delivery for MJDF will be clustered in families. The MJDF clients interviewed for this research understood MJD as a connection to their family story rather than specifically related to their genes. A standard appointment at a mainstream clinical genetic service will generally involve only the individual referred and one or two immediate family members. MJDF recognised that this is not the preferred or most effective method of discussing the familial nature of a genetic disease that has a devastating impact on families in many of these communities. This is evidenced by the interviews in this study where the majority of the community members affected by MJD indicated they preferred talking in family groups. Elsewhere, in response to the increasing recognition of the need to adapt traditional models of genetic counselling to provide more culturally informed services and overcome systemic barriers including geographical barriers, a range of alternative models are being developed including the option of group genetic counselling [56–62]. These models will become increasingly important in ensuring equitable clinical genetic services for diverse populations as health care moves increasingly into an era of precision medicine. With the emphasis on collective decision making, the option of including extended family/community members in consultations may also be relevant for other specialist health services that are operating in remote Aboriginal communities.

By incorporating the principles from the MJDF model and taking a community-based, person-and family-centred approach, mainstream specialist services including clinical genetics services may improve engagement with Aboriginal Australians and provide more comprehensive care. Continued engagement within communities is an essential first step in designing client led service delivery. Rather than providing services that are rigid and bound by the restrictions of a jurisdictional mandate, services must be flexible enough to accommodate diversity of Aboriginal Australians cultural values and adapt at a local community level. Central to this is creating a clinical setting where clients feel culturally safe and therefore more willing to interact with services [62]. Mainstream services can achieve this through consultations on country and in community, employing local Aboriginal workers and through implementation of cultural safety guidelines [18]. By building on established community organisations with pre-existing and productive relationships within the community, mainstream services can achieve a person-and family-centred delivery across a range of different contexts. Partnerships with organisations such as MJDF or ACCHSs embedded within the communities may help facilitate the building of genuine relationships and trust between specialist service providers and the communities they serve where high staff turnover and infrequent visits may otherwise limit the depth of these interactions [9, 63]. Such partnerships foster an exchange of information enabling the delivery of services to be shaped by the locality of its users. This will in turn aid in the development of a local workforce already familiar with the speciality service itself [64]. The success of MJDF in providing effective specialist treatment in remote community settings where mainstream services have been insufficient to meet the needs of the population, shows us that the key aspects from this community-based, person- and family-centred model has broad application in the equitable delivery of specialist health services.

## Supporting information

S1 File (DOCX)

S2 File (DOCX)

S3 File (DOCX)
