# Supplementary material for: A community-based co-designed genetic health service model for Aboriginal Australians
Source: PLoS One. 2020 Oct 29;15(10):e0239765. doi: 10.1371/journal.pone.0239765 (PMC7595342; doi:10.1371/journal.pone.0239765)
Supplement: S1 File — (DOCX) [file pone.0239765.s001.docx]

**Interview Protocol**

**For MJDF Consumers / Guardians /Carers**

(clients who have attended one of the clinical genetic service providers involved in project)

The patient journey mapping gives MJDF clients/carers/guardians, the opportunity to share their story with a focus on genetic services that have been utilised.

*The interviewer will begin by discussing the purpose of the research study, talking through the ‘plain language statement’ and ensuring they have given informed consent to participate. They will then gain verbal consent to begin audio recording.*

***Emphasise that this research is about the MJD being in your family and that lots of people in your family have it. What you know and feel about that.***

- Thank you <name> for helping us with this research. Did you grow up here?
- What are some of the things you like here? Things you like doing?
- **If it’s ok, tell me your story about MJD?**
- What do you think about when I say that word “genetics”? What does it mean?
- Why do you think lots of your family have MJD?
- Who told you that MJD story of it being passed down in the family?
- How did it make you feel? *(finding about that MJD story of being passed down).*
- Who do you feel safe talking about that MJD family story with?
- What makes you strong in your MJD story? (s*trong in your mind or heart*)

*I’ve got some pictures here of different things and places that have something to do with your MJD story. Do you think we could go through these pictures and you tell me how you feel or what you think when you go to these places or do these things?*

*PICS:*

***1) Home/community***

- Do you talk about MJD with your family? Does that help? How does it make you feel?

***2) Primary Health Care Clinic***

- How do you get to the clinic?
- Does anyone at the clinic every talk to you about “genetics”? *(or that MJD is passed down in the family)*

***3) Communication of MJDF “genetic themed” visit to community*** *(Notification of genetic service provider appointment being booked)*

- How do you find out when [genetic specialist] are coming to community [name of place]?
- When you know they are coming, and that they will talk about ‘genetics,’ and MJD being passed down in the family, how do you feel How does it make you feel?

***4) MJDF “genetic themed” visit to community*** *(Genetic services appointment)*

- Do you remember watching the videos the MJD mob have about passing on MJD in families?
  - Is this a good way to understand the story
  - *Do you like learning about these genes?*
  - *OR Can you tell me what you understood from watching the videos??*
- When you talk together with the MJD mob about your family tree:
  - How does it make you feel?
  - Is it a good way to tell your family story? *(Having it on paper who has MJD in your family)*
- Do you like talking to the MJDF mob by yourself or with your family with you all together?
- Does it help talking to other people about this MJD?
  - If yes:
- What is the proper way for you to talk about family business like MJD? *(who are the right people to talk to? one person or many? when is the right time to talk about it?)*

***5) Follow up specialist care & travel***

- Have you ever needed to go to a doctor appointment away from <community>?
  - If yes:
    - Can you tell me about that?
- Did the MJD mob help?
- Do you think it would have been harder without the MJD mob or ok?

**Overall**

- What is hardest about having out that MJD is in your family?
- Is there anything that makes having MJD in your family easier?
- Is there anything else that is important to you that we haven’t talked about?
  - Would understanding these things make talking about your family’s story about MJD better?
