# Supplementary material for: A community-based co-designed genetic health service model for Aboriginal Australians
Source: PLoS One. 2020 Oct 29;15(10):e0239765. doi: 10.1371/journal.pone.0239765 (PMC7595342; doi:10.1371/journal.pone.0239765)
Supplement: S2 File — (DOCX) [file pone.0239765.s002.docx]

**Interview Guide**

**For Clinical Genetic Service (CGS) Providers**

This interview guide has been adapted to be applicable to MJD staff.

*The interviewer will begin by discussing the purpose of the research study, talking through the ‘plain language statement’ and ensuring they have given informed consent to participate. They will then gain verbal consent to begin audio recording.*

- So <name>, how would you describe your role working with the MJDF?
- Could you define who you and the Foundation more broadly, provides services for?
- Could you please describe any training you had before starting work with the Foundation?

*MJD is fully penetrant, that is 100% of individuals with the genetic mutation develop the disease, and is also autosomal dominant, that is an individual with MJD has 50 percent chance passing on the genetic mutation/disease to their children.*

- Is the genetic nature of MJD (as described above) ever brought up with you by clients? If so, how do you respond?
- More generally, does the genetic nature of MJD (as described above) influence your interactions with clients or potential clients? (*If prompts needed - impact on families or early monitoring for onset of disease)*

**Primary questions:**

1. **In general, do you think the MJDF clients/families you see are interested in learning more about the genetic nature of MJD (as described above)?**
2. **Do you think that the options that are available to determine if someone carries the MJD gene should be conveyed? And if so, to whom; those affected by MJD? Those at risk of MJD? Or as many community members as possible where there is a high prevalence of MJD?**
   - **Do you have any insights or examples about how this is best done?**

*I now want to ask you a bit more about the services the MJDF offer more broadly with an emphasis on genetic aspects*

- Could you comment on the following aspects of care for services you offer MJDF clients/families, including how you try to ensure each of these are addressed:

1. Affordability
2. Accessibility – *include where you don’t go and why*
3. Accommodation & Acceptability – *how well the services provide flexibility to meet the needs of clients and whether the services are offered in an environment that is appropriate for the client? Eg. Provision for varying literacy levels, language barriers, cultural considerations and differences in the understanding of health and illness among Indigenous people compared to non-Indigenous.*

- Do the MJDF clients know when there is a “genetic themed” visit to their particular community planned? If yes, how is this communicated to them?
- Do you notice more interest amongst the MJDF clients in genetics after a “genetic themed” visit to the communities? If so, how long does this interest generally last?
- What do you think is most challenging in ensuring a high quality, culturally appropriate and accessible services for Aboriginal and/or Torres Strait Islander people?
- Broadly speaking, what are your views on how well services and care within the NT is coordinated in the current health care system for Aboriginal people who are believed to have a genetic disorder or susceptibility?
  - And how do you see MJDF services fitting into, or working with, the mainstream services.
- Finally, can you think of any strategies that you are happy to share, that you have found successful in maximising engaging of MJDF clients/families?
