# Supplementary material for: A community-based co-designed genetic health service model for Aboriginal Australians
Source: PLoS One. 2020 Oct 29;15(10):e0239765. doi: 10.1371/journal.pone.0239765 (PMC7595342; doi:10.1371/journal.pone.0239765)
Supplement: S3 File — (DOCX) [file pone.0239765.s003.docx]

**Interview Guide**

**For Primary Health Care (PHC) Providers**

This interview guide has been adapted to be applicable to primary care staff working at Angurugu Health Centre on Groote Eylandt where there is a high prevalence of MJD and the MJDF has a permanent presence.

*The interviewer will begin by discussing the purpose of the research study, talking through the ‘plain language statement’ and ensuring they have given informed consent to participate. They will then gain verbal consent to begin audio recording.*

- So <name>, how would you describe your role at Angurugu Health Centre?
- How long have you been working for Angurugu Clinic? And what about working in this community or region?
- Do you identify as Aboriginal and/or Torres Strait Islander?
- Did you participate in any cultural competency or other training to help you understand the values and customs of the region before starting work here?
- What proportion of patients seen at the Angurugu Clinic identify as Aboriginal and/or Torres Strait Islander?
- Can you tell me about your understanding of genetic services?

**MJD section**

- Can you describe how you work with the MJD Foundation to maximise health outcomes for patients impacted by MJD?
- Symptoms arising from MJD differ between individuals and are diverse; from muscle weakness/spasms, fatigue, pain, nutritional, vision, sleep and continence issues. Given this variability, how do you see the role of primary care in ensuring that individuals with MJD are seen by the appropriate specialists?

*MJD is fully penetrant, that is 100% of individuals with the genetic mutation develop the disease, and is also autosomal dominant, that is an individual with MJD has 50 percent chance passing on the genetic mutation/disease to their children.*

- Given the genetic nature of MJD and the implications this has for entire families, what role, if any, do you think Angurugu Clinic as the primary care provider has in communicating this information?

**Other genetic conditions section**

- Is information about the referral pathway to clinic genetic services in the NT provided to staff at Angurugu Clinic?
- Apart from MJD, do you know of any patients seen at Angurugu Clinic that were suspected of having a genetic disorder or being at high risk of one?
- If YES to this question, follow Part A
- If NO to this question, follow Part B

**Part A**

- If you know the details, could you describe the details of how this patient(s) navigated the care pathway and the role of Angurugu Clinic including:
  - What information was provided to the patient when they presented at the clinic
  - Where they referred to clinical genetic services?
    - If yes, was a follow-up letter received and acted on?
  - Could you describe if they were monitored and supported by Angurugu Clinic, and if so how?
- Do you know whether patients presenting at Angurugu Clinic are routinely asked if “anything runs in the family” or a similar question? Or whether a pedigree of patients is taken, regardless of whether a genetic condition is suspected?
- Do you believe that there are any gaps or duplication in care for Aboriginal people who are believed to have a genetic disorder or susceptibility (or a condition requiring a specialist)? If so, what are they and can you speculate on why you think they may have occurred?
- Related to the above question, what are your views on how well services and care is coordinated in the NT health system for Aboriginal individuals who are believed to have a genetic disorder or susceptibility (or a condition requiring a specialist)?
- Are you aware of any patients who have sought out genetic services elsewhere? Eg another town, city or interstate?
- What intervention or program, if any, do you think would be useful in your community to help with genetic services?
- Would you like to know more about genetic services? If yes - how do you think this would best be delivered?

**Part B**

*Depending on the level of knowledge that the PHC provider has about clinical genetic services (judged by responses to earlier questions), explain what clinical genetic services are including:*

- *Clinical genetics is a medical specialty that provides education, "genetic counselling" and diagnostic services such as genetic testing, for individuals or families with, or at risk of, conditions that may have a genetic basis.*
- *Conditions are suspected of having a genetic basis when they run in biological families and don’t seem to have an environmental cause.*
- *Clinical genetic services aim to help those affected by, or at risk of, a genetic condition by reducing the impact of the condition on their quality of life.*
- How easy or difficult you think it would be to incorporate referrals and ongoing care and support for clients of this service that may have a genetic condition?
  - Could you please expand on the factors that would make it easy or difficult?
- Can you think of any genetic providers that service the region you work in?
- Are you aware of any clients who have sought out genetic services elsewhere? Eg another town, city or interstate?
- Do you know whether patients presenting at Angurugu Clinic are routinely asked if “anything runs in the family” or a similar question? Or whether a pedigree of patients is taken, regardless of whether a genetic condition is suspected?
- Do you believe that there are any gaps or duplication in care for Aboriginal people who are believed to have a genetic disorder or susceptibility (or a condition requiring a specialist)? If so, what are they and can you speculate on why you think they may have occurred?
- Related to the above question, what are your views on how well services and care is coordinated in the NT health system for Aboriginal individuals who are believed to have a genetic disorder or susceptibility (or a condition requiring a specialist)?
- What intervention or program, if any, do you think would be useful in your community to help with genetic services?
- Would you like to know more about genetic services? If yes - how do you think this would best be delivered?
